# Supplementary material for: Abnormal neuronal and synaptic morphology in Down syndrome brains reproduces in human isogenic cellular models
Source: Cell Death Dis. 2026 Jun 10;17(1):730. doi: 10.1038/s41419-026-08956-y (PMC13478479; doi:10.1038/s41419-026-08956-y)
Supplement: Supplementary file 1 [file 41419_2026_8956_MOESM10_ESM.pdf]

# WB MEMBRANES

# FIGURE 1

# D21

TUBB3 (55kDa)

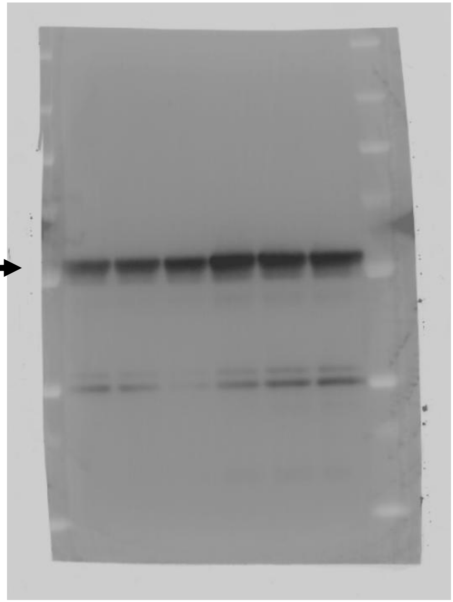

ß -actin (42kDa)

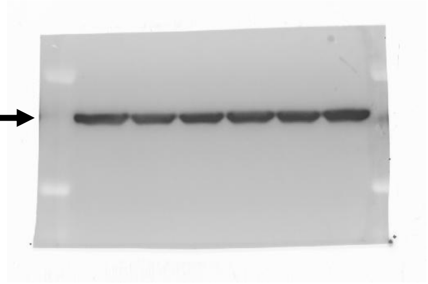

LEGEND:
Lines 1, 2, 3 – D21 DIV40
Lines 4, 5, 6 – D21 DIV60

# T21

TUBB3 (55kDa)

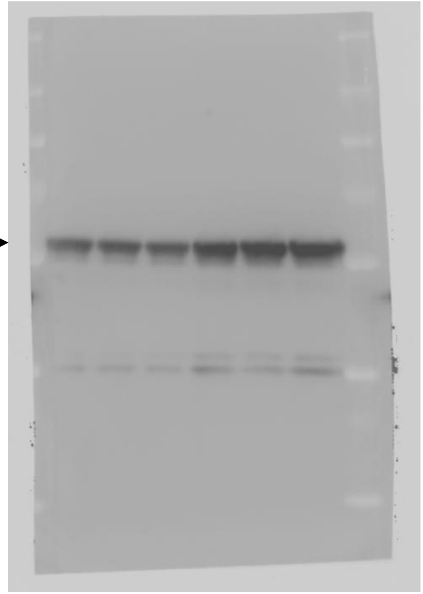

ß -actin (42kDa)

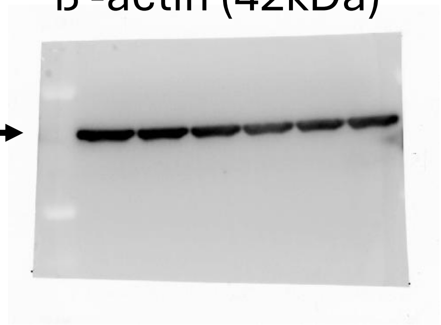

LEGEND:
Lines 1, 2, 3 – T21 DIV40
Lines 4, 5, 6 – T21 DIV60

# FIGURE 2

# D21

GFP (27kDa)

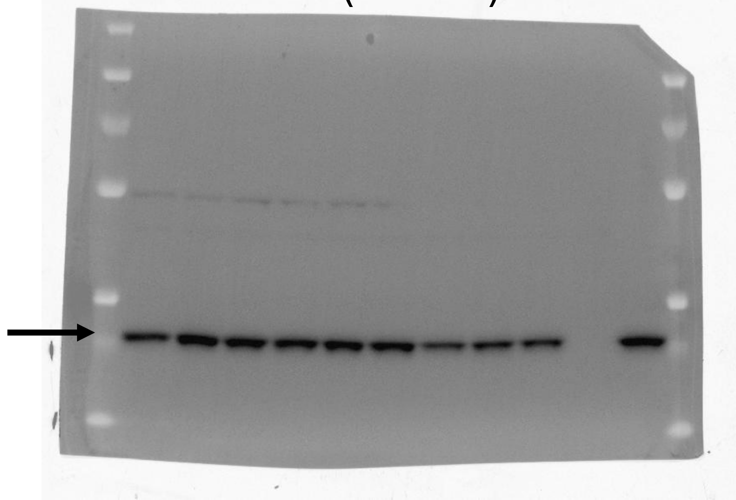

ß -actin (42kDa)

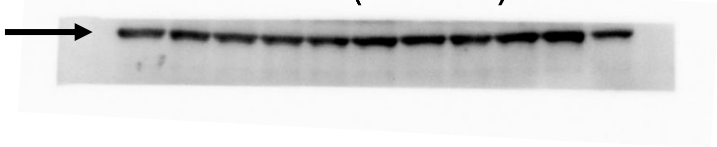

LEGEND:
Lines 1, 2, 3 – D21 DIV70
Lines 4, 5, 6 – D21 DIV100
Lines 7, 8, 9 – D21 DIV130
Line 10 – D21 hCSs DIV100
Line 11 – D21 hStrSs DIV100

# T21

GFP (27kDa)

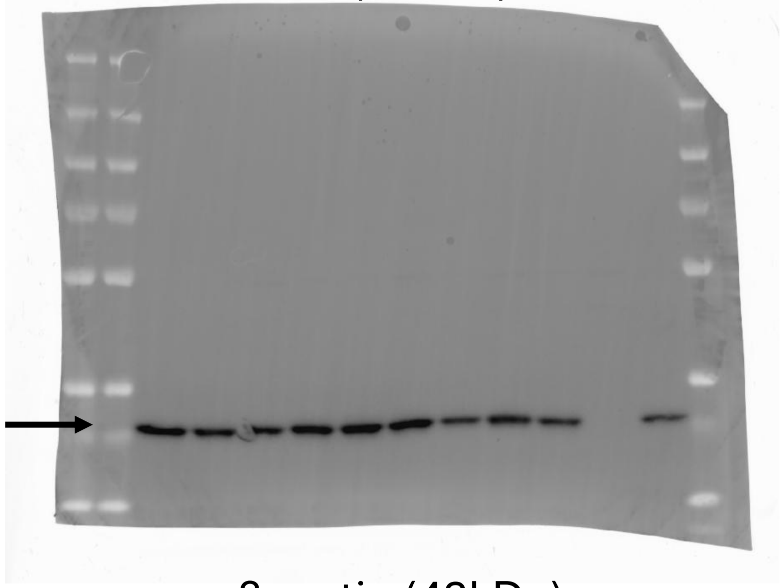

ß -actin (42kDa)

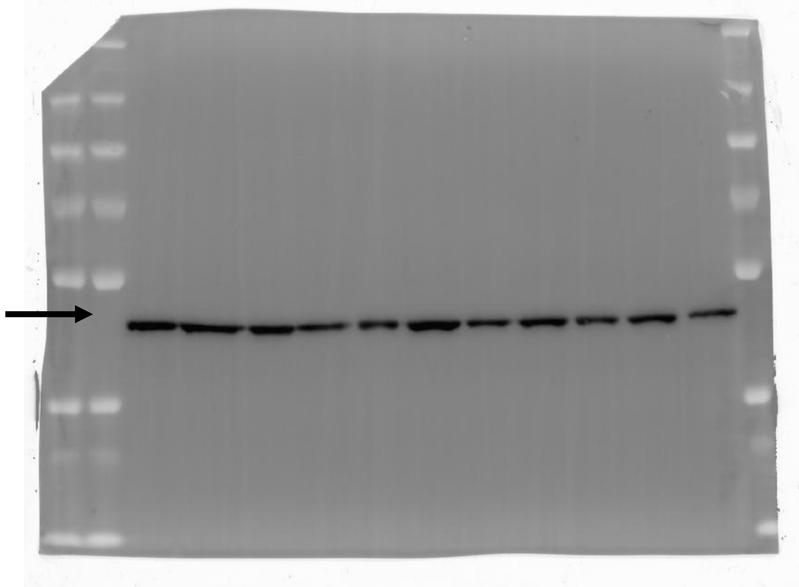

LEGEND:
Lines 1, 2, 3 – T21 DIV70
Lines 4, 5, 6 – T21 DIV100
Lines 7, 8, 9 – T21 DIV130
Line 10 – T21 hCSs DIV100
Line 11 – T21 hStrSs DIV100

# D21

TUBB3 (55kDa)

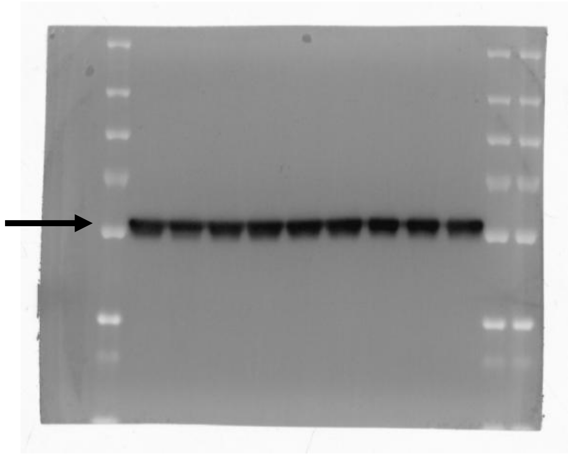

LEGEND:
Lines 1, 2, 3 – D21 DIV70
Lines 4, 5, 6 – D21 DIV100
Lines 7, 8, 9 – D21 DIV130

ß -actin (42kDa)

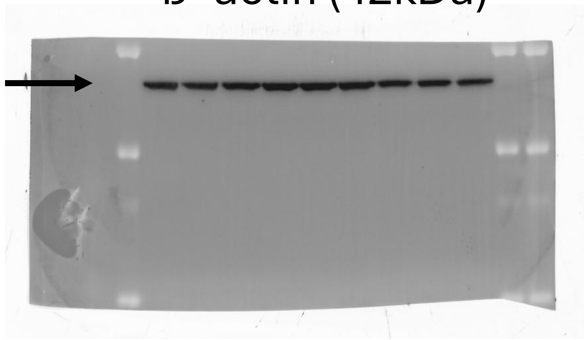

# T21

TUBB3 (55kDa)

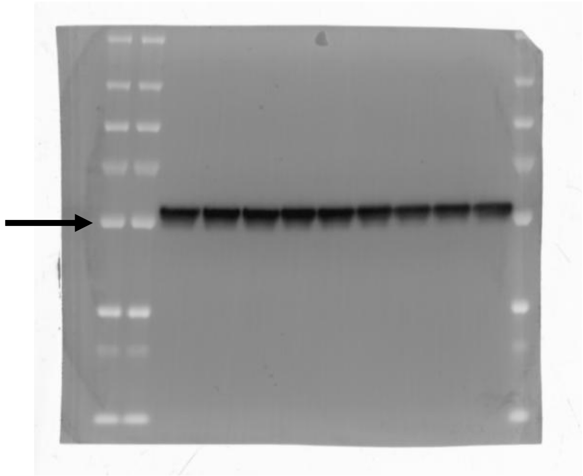

LEGEND:
Lines 1, 2, 3 – T21 DIV70
Lines 4, 5, 6 – T21 DIV100
Lines 7, 8, 9 – T21 DIV130

ß -actin (42kDa)

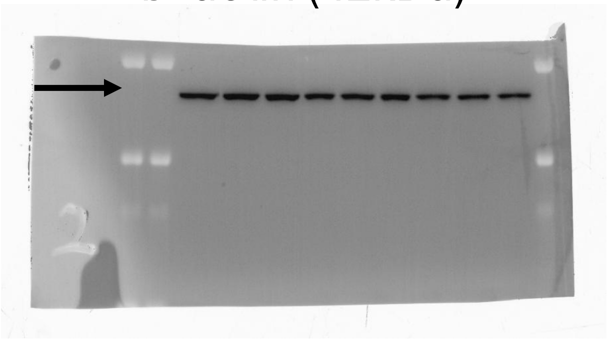

FIGURE 4

# D21

AIF (67kDa)

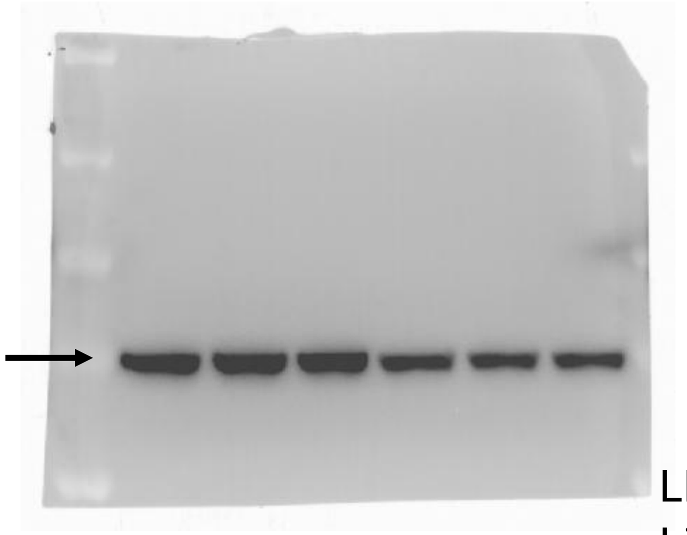

LEGEND:
Lines 1, 2, 3 – D21 C3
Lines 4, 5, 6 – D21 C7

ß-actin (42kDa)

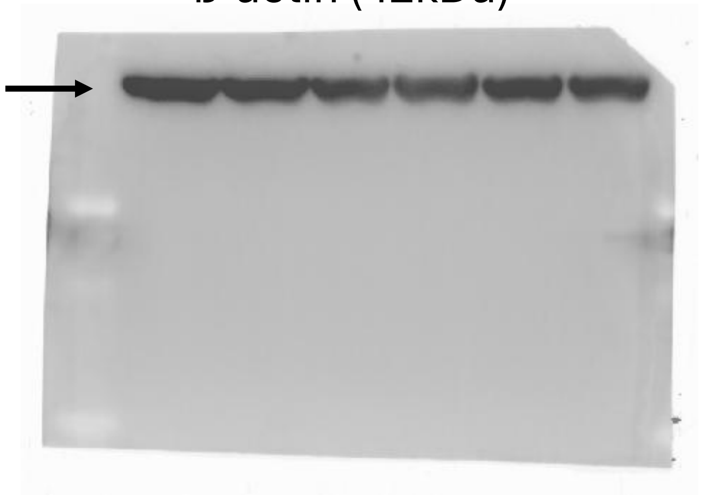

# T21

AIF (67kDa)

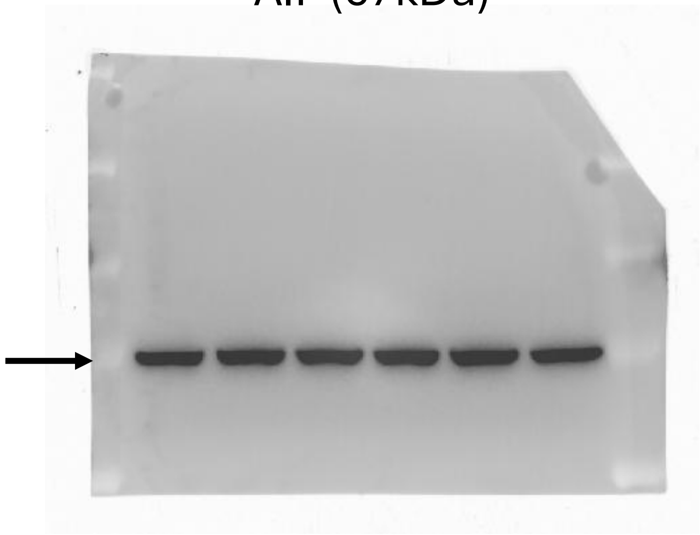

LEGEND:
Lines 1, 2, 3 – T21 C6
Lines 4, 5, 6 – T21 C5

ß -actin (42kDa)

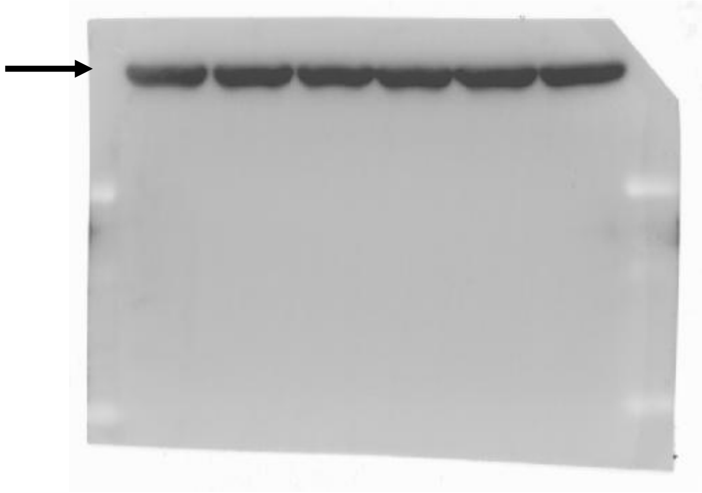

# FIGURE 5

# D21

PSD95 (95kDa)

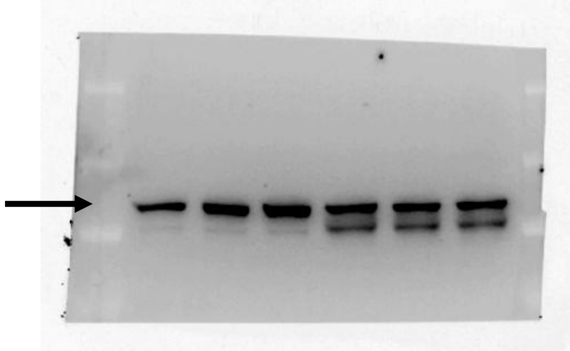

LEGEND:
Lines 1, 2, 3 – D21 DIV40
Lines 4, 5, 6 – D21 DIV60

ß -actin (42kDa)

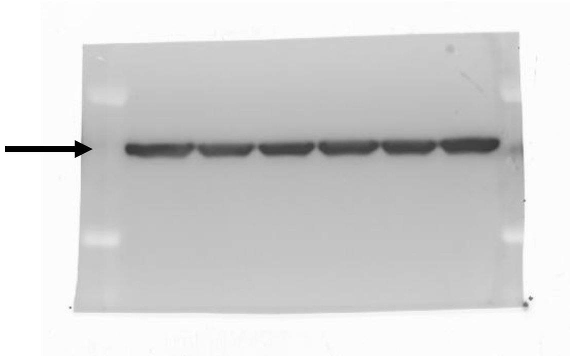

# T21

PSD95 (95kDa)

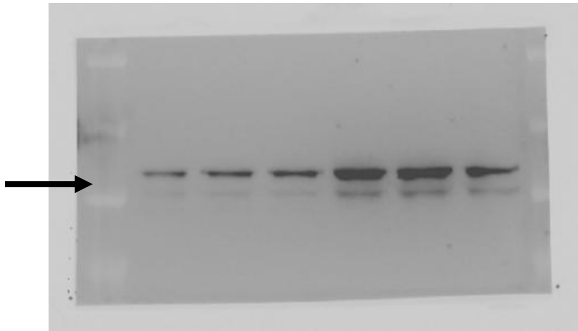

LEGEND:
Lines 1, 2, 3 – T21 DIV40
Lines 4, 5, 6 – T21 DIV60

ß -actin (42kDa)

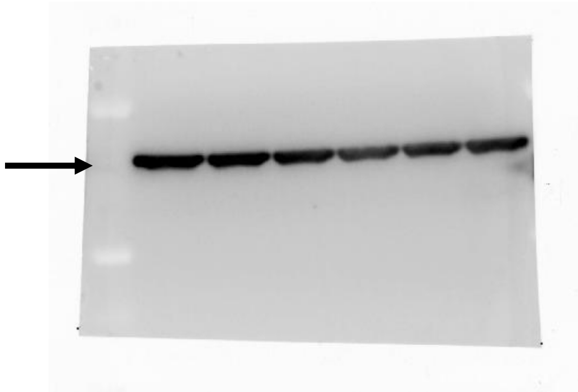

# D21

PSD95 (95kDa)

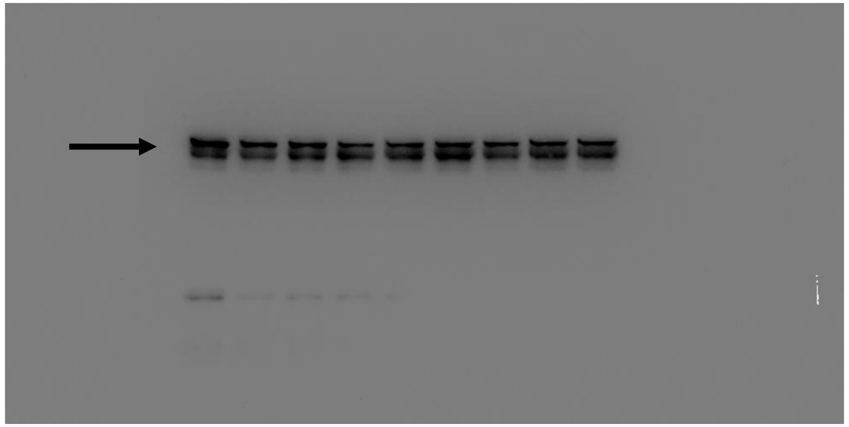

ß -actin (42kDa)

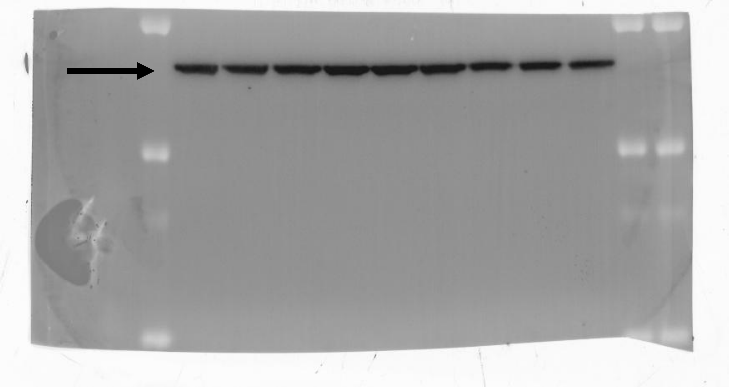

LEGEND:
Lines 1, 2, 3 – D21 DIV70
Lines 4, 5, 6 – D21 DIV100
Lines 7, 8, 9 – D21 DIV130

# T21

PSD95 (95kDa)

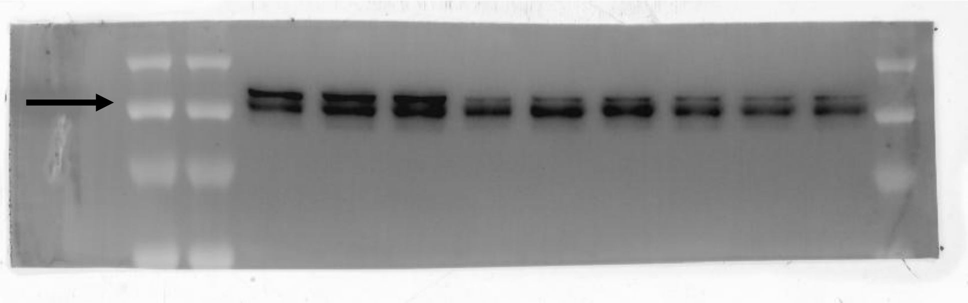

ß -actin (42kDa)

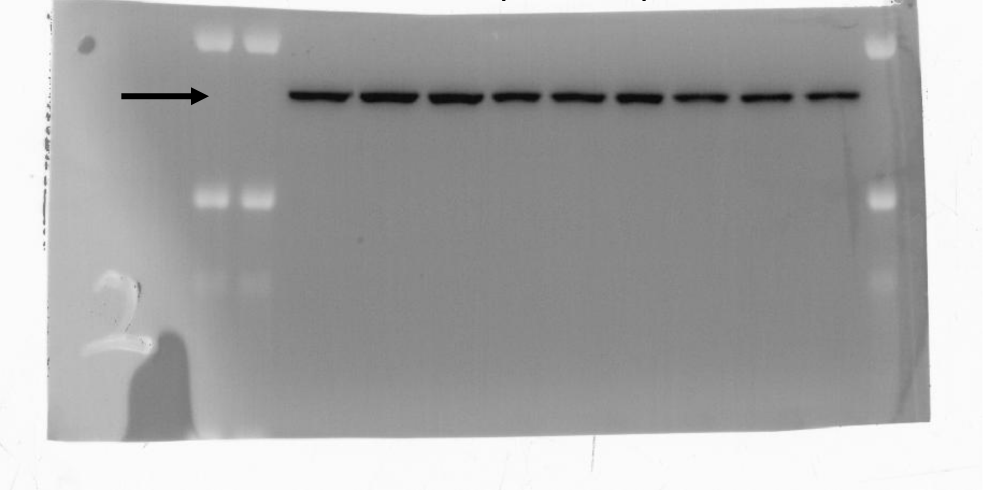

LEGEND:
Lines 1, 2, 3 – T21 DIV70
Lines 4, 5, 6 – T21 DIV100
Lines 7, 8, 9 – T21 DIV130

# FIGURE 7

# D21

SOX2 (37kDa)

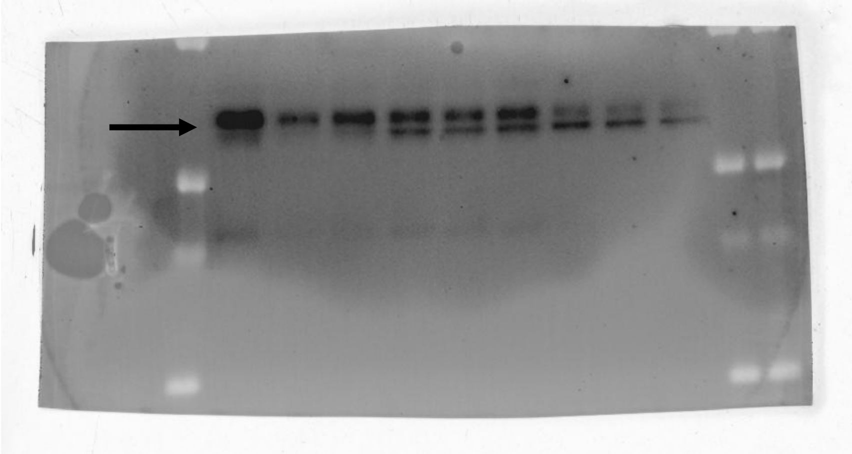

ß -actin (42kDa)

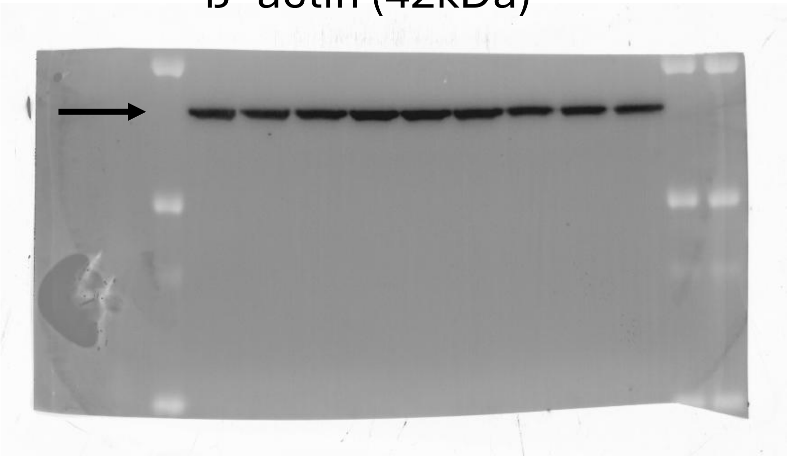

LEGEND:
Lines 1, 2, 3 – D21 DIV70
Lines 4, 5, 6 – D21 DIV100
Lines 7, 8, 9 – D21 DIV130

# T21

SOX2 (37kDa)

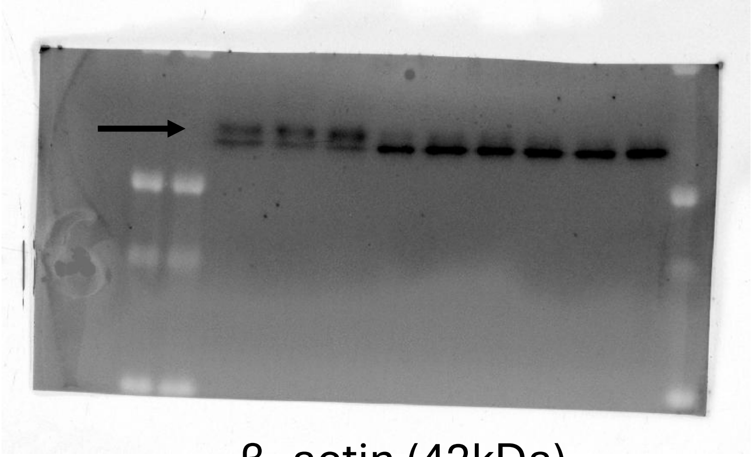

ß -actin (42kDa)

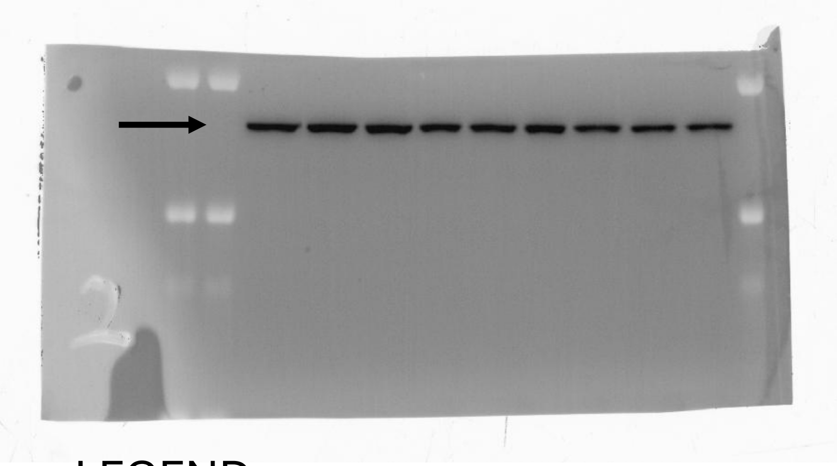

LEGEND:
Lines 1, 2, 3 – T21 DIV70
Lines 4, 5, 6 – T21 DIV100
Lines 7, 8, 9 – T21 DIV130

# SUPPLEMENTARY FIGURE 4

# D21

GEPH (93kDa)

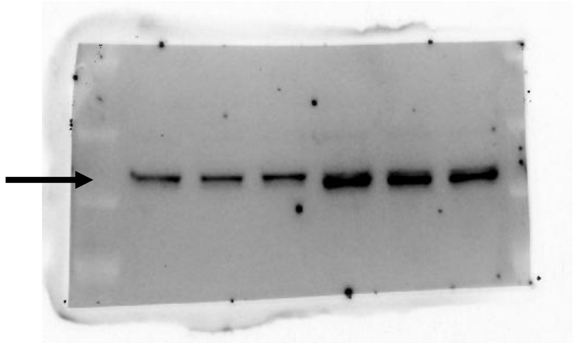

ß -actin (42kDa)

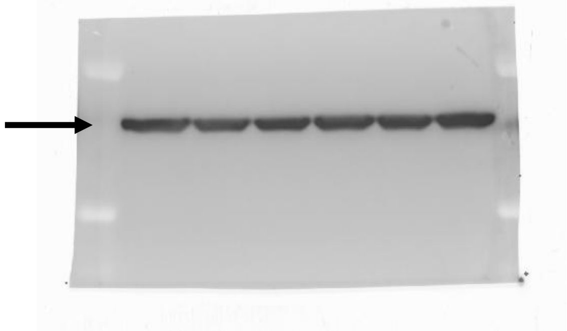

LEGEND:
Lines 1, 2, 3 – D21 DIV40
Lines 4, 5, 6 – D21 DIV60

# T21

GEPH (93kDa)

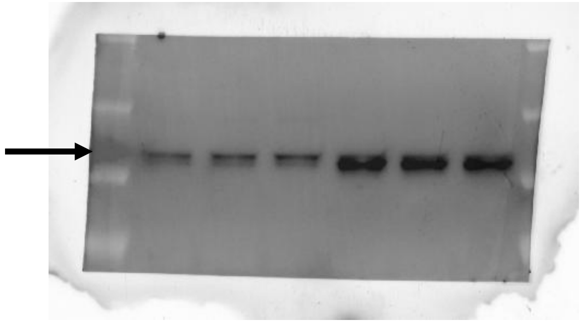

ß -actin (42kDa)

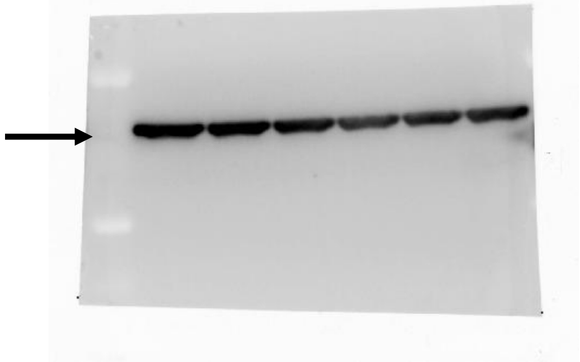

LEGEND:
Lines 1, 2, 3 – T21 DIV40
Lines 4, 5, 6 – T21 DIV60

# D21

GEPH (93kDa)

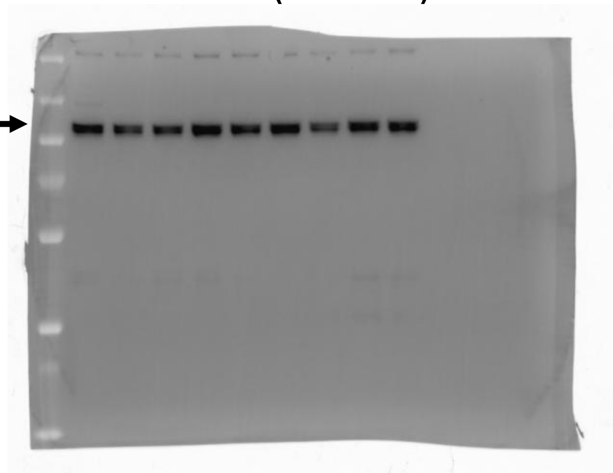

ß -actin (42kDa)

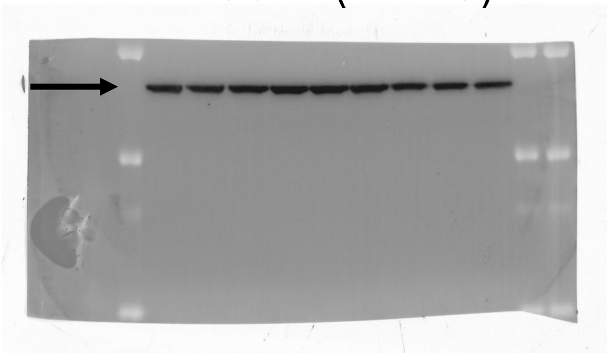

LEGEND:
Lines 1, 2, 3 – D21 DIV70
Lines 4, 5, 6 – D21 DIV100
Lines 7, 8, 9 – D21 DIV130

# T21

GEPH (93kDa)

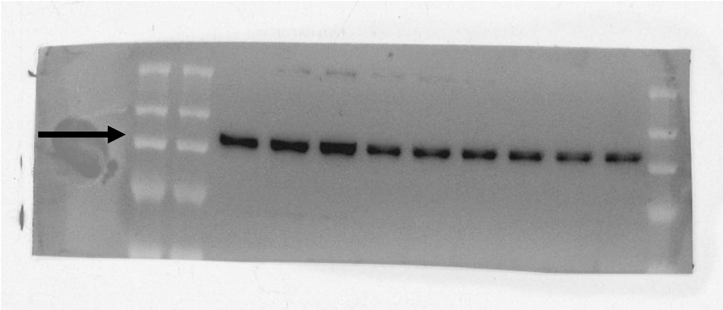

ß -actin (42kDa)

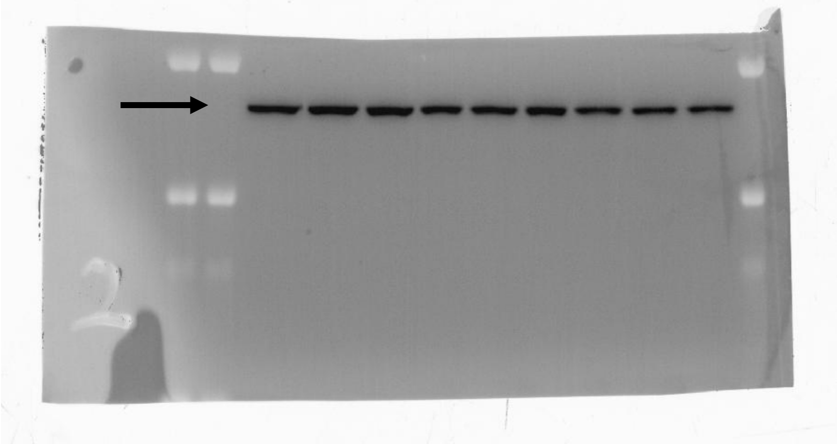

LEGEND:
Lines 1, 2, 3 – T21 DIV70
Lines 4, 5, 6 – T21 DIV100
Lines 7, 8, 9 – T21 DIV130

# SUPPLEMENTARY FIGURE 5

# D21

SYN-1 (77kDa)

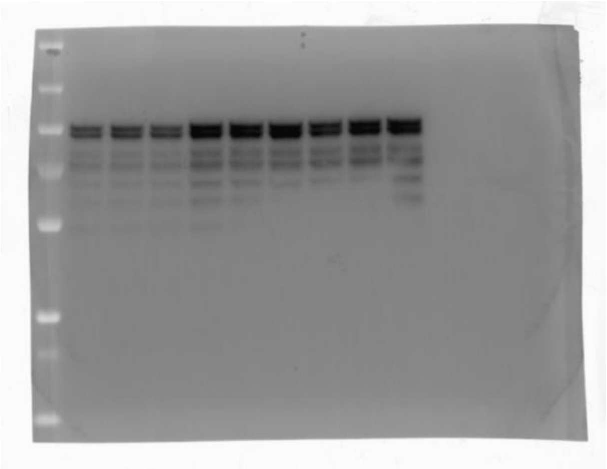

ß -actin (42kDa)

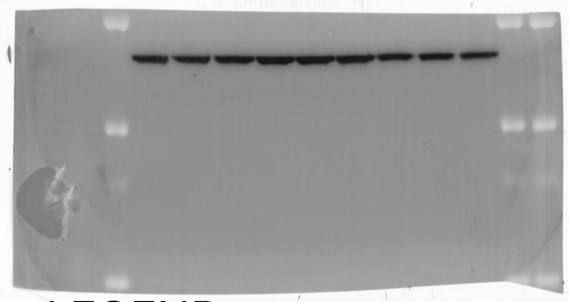

LEGEND:
Lines 1, 2, 3 – D21 DIV70
Lines 4, 5, 6 – D21 DIV100
Lines 7, 8, 9 – D21 DIV130

# T21

SYN-1 (77kDa)

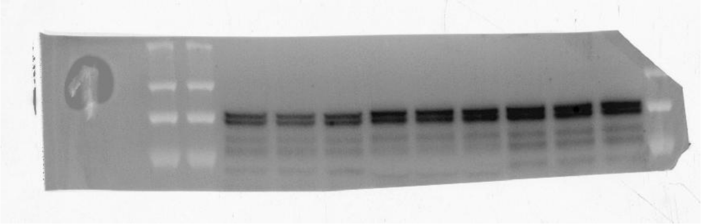

ß -actin (42kDa)

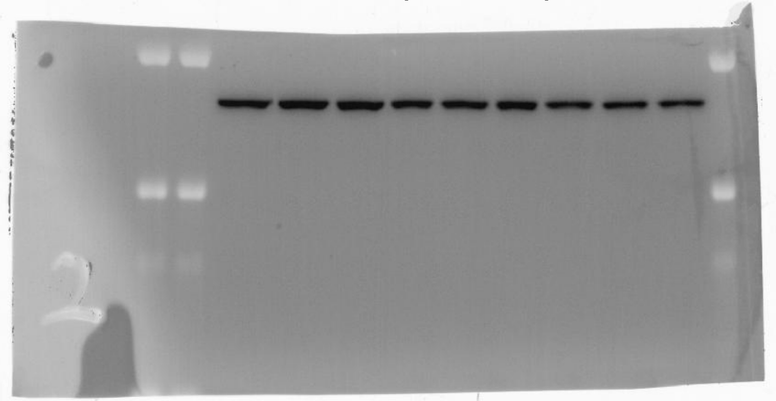

LEGEND:
Lines 1, 2, 3 – T21 DIV70
Lines 4, 5, 6 – T21 DIV100
Lines 7, 8, 9 – T21 DIV130
